# Development and validation of the self-consciousness type scale

**DOI:** 10.3389/fpsyg.2024.1362324

**Published:** 2024-07-05

**Authors:** Jiye Lee, Hyemi Baek, Eunjee Oh, Jin-young Kim, Young-gun Ko

**Affiliations:** ^1^School of Psychology, Korea University, Seoul, Republic of Korea; ^2^Department of Child Studies, Seoul Women’s University, Seoul, Republic of Korea

**Keywords:** self-consciousness, self-consciousness type, growth-oriented self-consciousness, defensive self-consciousness, regulatory focus, promotion-focused self-consciousness, prevention-focused self-consciousness

## Abstract

**Introduction:**

Previous research has highlighted the duality of self-consciousness, which simultaneously plays adaptive and maladaptive roles. This study aims to develop a measure that categorically distinguishes between different types of self-consciousness styles based on the Regulatory Focus Theory (RFT) and examines their relationship with mental health-related indicators.

**Methods:**

Data were gathered through an online mental health survey conducted at a University Student Counseling Center in Seoul. The study involved exploratory factor analysis, confirmatory factor analysis, and reliability and validity analysis, which resulted in the development of a 14-question Self-Consciousness Type Scale (SCTS).

**Results:**

Both exploratory and confirmatory factor analyses validated the two-factor structure of the SCTS. The fit indices of the final model indicated a good fit, with high internal consistency for both sub-factors. Convergent and discriminant validity were confirmed through correlations between the sub-scales. Cluster analysis identified four distinct subtypes of self-consciousness styles: Growth-oriented, Defensive, Ambivalent, and Low-focus self-consciousness. Group difference analysis revealed significant differences in mental health-related variables among the subtypes, supporting the 2 × 2 model of prevention-focused and promotion-focused self-consciousness.

**Discussion:**

The findings support the SCTS as a valid measurement tool capable of distinguishing four distinct types of self-consciousness, aligning with the multidimensional model of self-consciousness. The study’s limitations and implications were discussed based on the results, emphasizing the potential applications of the SCTS in mental health research and practice.

## 1 Introduction

Humans are the only animals that can think and pay conscious attention to the abstract idea of ‘self’ (Leary and Buttermore, 2003; Rochat, 2018). In their Objective Self-Awareness Theory, Duval and Wicklund (1972) introduced the concept of self-awareness, the act of recognizing and contemplating oneself as an object of attention. They asserted that human attention possesses bidirectionality, directed towards either the external environment or one’s internal self. Ingram (1990) coined the term self-focused attention to describe attention directed toward oneself. He defined it as “an awareness of self-referent, internally generated information that stands in contrast to an awareness of externally generated information derived through sensory receptors” (Ingram, 1990, p. 156). Through self-awareness, humans can perform higher-order cognitive functions such as self-reflection, processing external information in a self-referential manner, establishing self-identity, and taking the perspective of others (Bernstein and Davis, 1982; Howe and Courage, 1997). In particular, self-regulation, which regulates behavior by comparing one’s current state with the desired target state, has been emphasized as one of the fundamental functions of self-awareness (Duval and Wicklund, 1972; Carver and Scheier, 1990; Silvia and Duval, 2001).

If self-awareness represents a transient cognitive state where attention is focused inward, self-consciousness refers to a sustained propensity to think about oneself and maintain continuous self-focused attention (Fenigstein et al., 1975). In contrast to the fluctuating self-awareness contingent upon situations and contexts, self-consciousness, as a dispositional variable with individual differences, tends to exhibit relative stability within individuals and is scarcely influenced by measurement points or age factors (Davis and Franzoi, 1991). Consequently, research on self-focus has expanded beyond the existing paradigm of inducing temporary self-awareness states using external stimuli such as mirrors and examining the outcomes (Ickes et al., 1973; Scheier and Carver, 1977). It has evolved into a new trend that objectively measures an individual’s level of self-consciousness and explores its relationships with other psychological variables (Silvia et al., 2005). Empirical studies on dispositional self-consciousness have primarily been based on the model proposed by Fenigstein et al. (1975). They differentiated self-consciousness into private self-consciousness and public self-consciousness according to the aspects of oneself that an individual is primarily conscious of. Private self-consciousness refers to an individual’s tendency to focus on the internal aspects of the self, such as emotions, thoughts, and values, which are not readily apparent to others. Public self-consciousness pertains to an individual’s tendency to continuously scrutinize and direct attention towards outward expressions of self, such as appearance and behavior, considering how one is perceived by others. Fenigstein et al. (1975) developed the Self-Consciousness Scale (SCS) as a self-report measure to assess these aspects, which was initially developed and validated with a sample consisting of 243 undergraduate students.

The existing research on the relationship between self-consciousness and mental health has revealed that self-consciousness has a duality wherein it exhibits correlations with both psychological adaptation and maladaptation. This phenomenon is referred to as the ‘self-absorption paradox’ (Trapnell and Campbell, 1999). Primarily, self-consciousness involves adaptive facets, contributing to enhanced self-knowledge, the formation of sophisticated self-schemata, and the development of consistent and clear perceptions about oneself (e.g., Buss, 1980; Nasby, 1985; Trapnell and Campbell, 1999). Fenigstein et al. (1975) asserted that self-consciousness particularly predicts heightened responsiveness to insight-oriented psychotherapy, while Trudeau and Reich (1995) reported a positive correlation between private self-consciousness levels and psychological mindedness. Psychological mindedness denotes the capacity for reflecting on the meaning of one’s own and others’ behaviors, thoughts, and emotions, playing a role in promoting mental well-being and the efficacy of psychotherapeutic interventions (Farber, 1985). Moreover, Suls and Fletcher’s (1985) study revealed that heightened private self-consciousness serves as a protective factor in moderating the relationship between life stress and physical illness. Individuals with lower self-consciousness levels tend to exhibit a tendency to avoid stress rather than accepting and attentively examining psychological and physical reactions to stress, as compared to those with higher self-consciousness levels. Consequently, individuals with lower self-consciousness levels may be more susceptible to the possibility of stress leading to physical illness.

Meanwhile, numerous studies have addressed the pathological aspects of self-consciousness in contrast to its adaptive dimensions. According to Ingram’s (1990) review, a high level of self-focused attention serves as a transdiagnostic factor explaining a broad spectrum of psychopathology and maladaptation, including depression, anxiety, schizophrenia, suicide, and substance abuse. For instance, in the case of test anxiety, the shift of attention from external tasks to oneself hinders concentration on the task, leading to performance impairment and perpetuating a vicious cycle of heightened anxiety (Wine, 1971). Additionally, social anxiety tends to be perpetuated and exacerbated through a continuous monitoring process of oneself, driven by the fear of how others perceive one (Buss, 1980; Hope and Heimberg, 1988). Mor and Winquist (2002), through a meta-analysis, revealed a moderate-sized correlation between self-focus and negative affect (i.e., depression, anxiety, negative mood), a relationship consistently observed in both situationally induced self-awareness and dispositional self-consciousness. It is proposed that the heightened level of self-focus plays a role in eliciting or magnifying emotional distress, leading to chronic suffering. Consequently, some perspectives posit that alcohol abuse or suicidal tendencies may emerge as a means of escaping persistent self-attention (Hull, 1981; Baumeister, 1990).

The theories proposed to elucidate the paradox of self-consciousness can be categorized into two branches based on whether they perceive adaptive and pathological self-consciousness as two factors exhibiting continuity along a single dimension or as independent factors constituting qualitatively distinct dimensions (Lee and Kwon, 2005). One of the most influential models conceptualizing pathological self-consciousness coexisting within the same dimension as adaptive self-consciousness is the Self-Absorption Model introduced by Ingram (1990). This model posits that pathological self-consciousness, or self-absorption, is distinguished from normal self-focused attention in that it exhibits abnormalities in terms of degree, duration, and flexibility. Specifically, when one excessively engages in thinking about oneself, and this state persists for an extended period, becoming rigid and difficult to redirect attention elsewhere, normative self-focused attention becomes dysfunctional. According to this model, excessive self-focus leads to maladaptation; therefore, psychological interventions aimed at reducing self-focus can contribute to alleviating pathological symptoms (Ingram, 1990).

Other researchers have supported the multidimensional model of self-consciousness, positing that adaptive self-consciousness and maladaptive self-consciousness constitute qualitatively distinct dimensions (e.g., Creed and Funder, 1998; Trapnell and Campbell, 1999; Mor and Winquist, 2002). Mor and Winquist (2002) argued that the pathological effects of self-focus do not arise merely from a simple excess of self-focused attention or such tendencies. Instead, they proposed that it is valid to consider that different types of self-focus lead to varied outcomes depending on the context. For instance, when the context and nature of self-focus are not taken into consideration, self-focus tends to exacerbate the intensity of negative affect on average. However, self-focus following positive events did not exacerbate negative affect. Moreover, selectively attending to one’s positive aspects actually decreased negative affect (Mor and Winquist, 2002). This aligns with the concept of a depressive self-focusing style proposed by Pyszczynski and Greenberg (1987), who argued that individuals experiencing depression exhibit a characteristic self-awareness tendency. Specifically, they tend to focus more on themselves after negative events, while paying less attention after positive events. Such a tendency contributes to the maintenance and exacerbation of depressive symptoms, leading individuals into an aversive cycle of self-regulatory processes, reinforcing negative self-perceptions (Smith and Greenberg, 1981; Pyszczynski and Greenberg, 1987).

Trapnell and Campbell (1999), based on the Five-Factor Model of Personality (FFM), differentiated private self-consciousness into two factors activated by distinct motives: rumination and reflection. They posited that the inconsistency in the results across existing literature on self-consciousness arises from the coexistence of items assessing self-consciousness driven by disparate affective or motivational factors, such as negative affect (Ingram, 1989), the desire for self-knowledge (Franzoi et al., 1990), and the desire for uniqueness (Schlenker and Weigold, 1990), in the SCS (Fenigstein et al., 1975) utilized as a measurement tool. Trapnell and Campbell (1999) thus redefined self-consciousness activated by neurotic motives, such as negative affect and anxiety, as rumination, and self-consciousness activated by intellectual motives, such as curiosity, and related to openness to experience, as reflection. Furthermore, they found that rumination is primarily associated with pathological factors like anxiety and depression, while reflection correlates with neutral or adaptive factors. This model aligns with the study by Franzoi et al. (1990), which proposed heterogeneous motives underlying self-consciousness, namely the desires for self-knowledge and self-defense.

Meanwhile, questions regarding the validity and reliability of the Self-Consciousness Scale (SCS; Fenigstein et al., 1975), a tool predominantly used for measuring self-consciousness, have persistently been raised (Burnkrant and Page, 1984; Wicklund and Gollwitzer, 1987). Conceptually, Wicklund and Gollwitzer (1987) criticized the theoretical ambiguity regarding the operational definition of private and public self-consciousness and in differentiating between private and public self-consciousness. Particularly, they argued that public self-consciousness, rather than measuring self-attention, more validly assesses a degree of social sensitivity and responsiveness. In the case of private self-consciousness, inconsistencies have been noted, with simultaneous correlations observed with psychological health and maladaptive variables, as well as contradictory results in its association with variables such as social anxiety and paranoia (Monfries and Kafer, 1994; Smári et al., 1994). Lastly, attempts to validate the SCS across diverse groups have encountered challenges, as the two-factor structure proposed by Fenigstein et al. (1975) has not been consistently replicated. Additionally, the items of the SCS belonging to the public and private self-consciousness factors have varied slightly across studies (e.g., Creed and Funder, 1998; Trapnell and Campbell, 1999).

Against this backdrop, alternative tools have been developed to address the limitations of the SCS and to explain the paradox of self-consciousness. However, there is still insufficient consensus on the components that constitute self-consciousness and the measurement tools, as highlighted in a comprehensive review by DaSilveira et al. (2015). For instance, Christensen’s (1982) maladaptive self-consciousness scale (SCON) was developed to measure dysfunctional self-consciousness, but it is limited by its confinement to social situations, making generalization challenging. Trapnell and Campbell (1999) developed the Rumination and Reflection Questionnaire (RRQ), which measures rumination, the neurotic factor, and reflection, the factor related to openness. However, concerns have been raised about the validity of the RRQ in measuring self-consciousness, given its inability to significantly predict behavioral indicators of self-consciousness for both rumination and reflection factors (e.g., speed of self-related word retrieval in a word retrieval task, extent of using first-person pronouns in a sentence completion task), and the notion that rumination does not always involve awareness or thoughts about oneself (Papageorgiou and Wells, 2004; as cited in McKenzie and Hoyle, 2008) (Silvia et al., 2005).

In this study, we aimed to redefine the subfactors of self-consciousness based on Higgins’ Regulatory Focus Theory (Higgins, 1997; Higgins, 1998). According to Regulatory Focus Theory, self-regulation is driven by two motives: promotion and prevention, referred to as regulatory focus (Higgins, 1997). Goals guided by a promotion focus are associated with ideals, achievements, and growth, while goals guided by a prevention focus are associated with safety, duty, and responsibility. Consequently, individuals motivated by a promotion focus tend to behave in a manner that maximizes positive experiences, while those motivated by a prevention focus tend to act in a direction that prevents and minimizes negative experiences such as loss or risk. Considering that the two regulatory foci proposed in Regulatory Focus Theory exhibit differences in motivation derived from comparing the current self with the goal state, it can be anticipated that self-consciousness, serving as a ‘comparator’ in the self-regulation process by comparing the current self with the desired self (Carver and Scheier, 1981), would also operate differently based on whether it aligns with a promotion or prevention focus. Specifically, promotion-focused self-consciousness is expected to enhance positive experiences and move closer to the ideal self by thinking about oneself and paying attention. On the other hand, prevention-focused self-consciousness is assumed to avoid negative experiences that threaten self-esteem or are detrimental to oneself and to adhere to the criteria of the ought self without deviation.

Based on this, the present study assumes the existence of two independent dimensions of self-consciousness operating through promotion and prevention foci. The objective of the study is to develop a scale that categorically distinguishes four different types of self-consciousness by combining high and low levels of these two dimensions. Furthermore, through group difference analysis, the study aims to investigate whether the four identified subtypes show significant differences in mental health-related variables. The study follows three main theoretical premises. First, it supports the multidimensional model of self-consciousness, positing qualitative differences between adaptive and pathological self-consciousness (Trapnell and Campbell, 1999; Mor and Winquist, 2002; Lee and Kwon, 2005). Second, it adopts a motivational explanation suggesting that the distinct impacts of self-consciousness on psychological adaptation and maladaptation vary according to the primary motives for self-attention (Franzoi et al., 1990; Trapnell and Campbell, 1999). Lastly, considering the crucial role of self-consciousness in the self-regulation process (Baumeister, 1990; Mor and Winquist, 2002), the study assumes that self-consciousness operates under two regulatory foci: promotion and prevention focus.

## 2 Methods

### 2.1 Participants

We collected the data through an online survey conducted by a University Student Counseling Center in Seoul in May 2022. We recruited participants through e-mails and promotional materials posted on the Student Counseling Center website. All the participants voluntarily signed the online consent forms explicitly stating that survey results would be used anonymously for subsequent research aimed at improving mental health promotion programs. Additionally, all participants received individualized mental health reports reviewed by mental health professionals. We surveyed 2,291 undergraduate and graduate students and used the final data of 2,104 cases for analysis after excluding cases with incomplete or missing responses from the entire dataset. Among the included participants, the average age was 23.65 years (SD = 3.70), ranging from 18 to 54 years. Of the participants, 1,061 were female (50.4%), 1,038 were male (49.3%), and 5 participants (0.3%) chose genders other than male or female or chose not to specify their gender. In terms of educational background, the participants consisted of 1,607 undergraduate students (76.4%), 495 graduate students (23.5%), and 2 others (0.1%). We decided to use a college student sample in constructing and validating the SCTS, drawing from the previous research on the development of various self-report scales that measure self-consciousness (e.g., Fenigstein et al., 1975; Trapnell and Campbell, 1999). We conducted all data collection and analysis procedures following the approval of the Institutional Review Board (IRB).

### 2.2 Item selection for self-consciousness type scale

The SCTS was developed based on Higgins’ (1997) Regulatory Focus Theory, which presents two dimensions of regulatory focus operated by different motivations: promotion focus and prevention focus. Accordingly, the SCTS assumes two dimensions of self-consciousness driven by these distinct motivations. Additionally, the preliminary items of the SCTS were selected with reference to existing self-consciousness scales (e.g., Fenigstein et al., 1975). The specific scale items were developed through collaboration between two clinical psychology professors, grounded in the theoretical concepts. Consequently, an 18-question preliminary scale comprising promotion-focused and prevention-focused self-consciousness was created, each beginning with “I think a lot about myself because....” The participants are asked to indicate the extent to which each statement is like them on a 6-point Likert scale. After the initial development of the items, three graduate students specializing in clinical psychology conducted small group discussions to review and revise items with dual meanings or ambiguous wording. A preliminary analysis was then conducted using the 18 preliminary items, and factor analysis was performed to select the final items to be included in the scale.

### 2.3 Data analysis

We conducted a series of analyses to validate the scale, employing the split-sample approach, where one sample is randomly divided into multiple independent subsamples for EFA and CFA. Specifically, the entire sample was randomly divided into three subsamples in order to conduct preliminary analysis, exploratory factor analysis, and confirmatory factor analysis on each of the three independent samples, respectively. Reliability analysis, convergence and discriminant validity analysis, and between-group difference analysis were conducted on the total sample of 2,104 participants. The split-sample approach is commonly employed by researchers for model cross-validation and generalization purposes (e.g., Lorenzo-Seva, 2022). Floyd and Widaman (1995) stated that if the sample size is sufficiently large, dividing one sample into multiple subsamples for repeated validation may be more effective than conducting multiple analyses on the entire sample. Given that the total sample size in this study consisted of 2,104 participants, it was deemed sufficiently large for the split-sample approach.

Initially, we performed a factor analysis on the 18 preliminary questions to select scale items and assess their factor structure on Sample 1 (*N* = 215). Following that, we conducted an exploratory factor analysis on Sample 2 (*N* = 860) to extract latent factors. A confirmatory factor analysis was conducted on Sample 3 (*N* = 1,029) to validate the derived factor structure. The demographic characteristics (i.e., gender, age) of each sample are summarized in Table 1. To determine the number of participants assigned to each subsample, several guidelines were followed. First, we randomly selected approximately 10% of the total sample to conduct preliminary analysis for item selection following the guidelines of Treece and Treece (1982). The remaining sample was divided into two subsamples for exploratory factor analysis and confirmatory factor analysis. According to Kyriazos (2018), while the sample size has little impact on the statistical power of EFA, it significantly enhances the statistical power and accuracy of CFA. Therefore, we allocated a larger sample to CFA than to EFA, deeming it appropriate to enhance the robustness of our confirmatory analysis.

**Table 1:** Demographic features of the sample (*N* = 2,104).

Variable		Sample 1 (*N* = 215)		Sample 2 (*N* = 860)		Sample 3 (*N* = 1,029)		Total (*N* = 2,104)	
		Frequency	Percentage (%)	Frequency	Percentage (%)	Frequency	Percentage (%)	Frequency	Percentage (%)
Gender	Male	118	54.9	410	47.7	510	49.6	1,038	49.3
	Female	97	45.1	449	52.2	515	50	1,061	50.4
	Other	–	–	1	0.1	4	0.4	5	0.3
Age	10–19	26	12.1	61	7.1	76	7.4	163	7.7
	20–29	178	82.8	749	87.1	887	86.2	1814	86.2
	30–39	9	4.2	46	5.3	57	5.5	112	5.3
	40–49	2	0.9	4	0.5	8	0.8	14	0.7
	50–59	–	–	–	–	1	0.1	1	0.1
	*M* (SD)	23.58 (3.85)		23.57 (3.55)		23.74 (3.80)		23.65 (3.70)	

We checked reliability by assessing internal consistency for the entire scale and its sub-scales, and the item-total correlation of the scale. To this end, correlations between the newly developed scale and the widely used Self-Consciousness Scale (SCS), which serves as a representative measure of self-consciousness, were first examined to assess whether the newly developed scale adequately reflects self-consciousness tendencies. Additionally, to assess criterion-related validity, correlations were investigated between self-consciousness and representative indicators closely associated with psychological adaptation in previous studies such as self-esteem (e.g., Ickes et al., 1973), psychological well-being (e.g., Lyke, 2009), and college adjustment (LaBrie et al., 2008). Furthermore, correlations with representative indicators of psychological maladaptation and pathological characteristics including depression (e.g., Smith and Greenberg, 1981; Pyszczynski et al., 1991), anxiety (e.g., Mor and Winquist, 2002; Spurr and Stopa, 2002), shame (e.g., Crozier, 1998), suicidal ideation (e.g., Baumeister, 1990; Schaller, 1997), and perceived stress (e.g., Mullen and Suls, 1982), were also investigated.

Additionally, we conducted a cluster analysis using the k-means clustering method (Lange et al., 2002) to classify participants into distinct self-consciousness types. Based on theoretical background, interpretability, and coefficients in the agglomeration schedule, we determined that the optimal number of clusters was four. For the analysis, we used the standardized z-scores of two SCTS subscales. After classifying the participants into four distinct groups, we conducted a one-way ANOVA to explore significant differences in mental health-related variables between the groups. We designated the group with high levels of both promotion-focused and prevention-focused self-consciousness as the ambivalent self-consciousness group. We termed the group with high promotion-focused but low prevention-focused self-consciousness as the growth-oriented self-consciousness group. We dubbed the group with low promotion-focused and high prevention-focused self-consciousness the defensive self-consciousness group. Finally, we named the group with low self-consciousness in both promotion and prevention focus the low-focus self-consciousness group. We used IBM SPSS Statistics 27 for conducting descriptive statistics, exploratory factor analysis, reliability, validity analysis, cluster analysis and difference analysis between groups. Additionally, we used MPlus 8.7 for performing confirmatory factor analysis.

### 2.4 Measures

#### 2.4.1 Self-consciousness scale

We utilized the Self-Consciousness Scale (SCS) developed by Fenigstein et al. (1975) and translated into Korean by Lee (1988). The Cronbach’s alphas measured in the present study were 0.80 for the public self-consciousness subscale, 0.65 for the private self-consciousness subscale, and 0.84 for the social anxiety subscale.

#### 2.4.2 Korean version of the center for epidemiologic studies depression scale (K-CES-D)

We used the Center for Epidemiologic Studies Depression Scale developed by Radloff (1977) and translated and validated in Korean by Cho and Kim (1993). Consisting of a total of 20 items, the scale measures depressive symptoms of the participants based on the Diagnostic and Statistical Manual of Mental Disorders (DSM). The K-CES-D is scored on a scale from 0 to 60. According to previous research, a score of 16 or higher indicates mild depression, while scores between 16 and 24 are evaluated as moderate depression. A score of 25 or higher is classified as major depression, requiring professional counseling and treatment. The Cronbach’s α measured in the present study was 0.94.

#### 2.4.3 Korean version of generalized anxiety disorder−7

We used the Korean version of the Generalized Anxiety Disorder-7 (K-GAD-7), initially developed by Spitzer et al. (2006) and validated in Korea by Seo et al. (2014). This scale is a 7-item measure developed to assess general anxiety symptoms. The scores range from 0 to 21, with higher scores indicating greater severity of symptoms. Previous studies use a score of 5 as the cutoff for mild anxiety. We measured a Cronbach’s α of 0.90 for the scale.

#### 2.4.4 Rosenberg self-esteem scale

We used Rosenberg’s Self-Esteem Scale (RSES) developed by Rosenberg (1965) and translated into Korean by Jeon (1974). It consists of 10 items that measure self-esteem. The Cronbach’s α of the scale measured in the present study was 0.90.

#### 2.4.5 Korean version of the experience of shame scale

We used the Characterological Shame subscale of the Korean version of the Shame-Experience Scale (K-ESS). The K-ESS was developed by Andrews et al. (2002) and validated by Sin et al. (2015) in Korea. In the present study, we used nine questions of the characterological shame factor to measure the degree of experiencing overall shame about one’s personal habits, attitudes, personality, and abilities. Cronbach’s α of the scale measured in the present study was 0.89.

#### 2.4.6 Korean version of the Beck scale for suicide ideation

We used the Korean version of the Beck Suicide Ideation Scale (K-BSS), initially developed by Beck and Steer (1993) and translated and validated in Korean by Choi et al. (2020). The scale consists of 19 items developed to measure the presence and severity of suicidal ideation. The score ranges from 0 to 38. Previous research indicates that a score of 8 or below suggests no suicidal ideation, while scores between 9 and 11 indicate a higher-than-average level of suicidal thoughts for the age group. Scores between 12 and 14 suggest a significantly elevated level of suicidal thoughts compared to the age group and may necessitate psychiatric counseling. A score of 15 or above indicates a level of suicidal ideation that poses a real risk of suicidal behavior, requiring in-depth counseling. The Cronbach’s α of the scale measured in the present study was 0.87.

#### 2.4.7 Korean version of the perceived stress scale

We used the Perceived Stress Scale (PSS) developed by Cohen et al. (1983) and translated and validated in Korean by Park and Seo (2010). It consists of a total of 14 items that measure the subjective perception of stress. In the present study, we found Cronbach’s α of the scale to be 0.85.

#### 2.4.8 College adjustment inventory-short form (CAI-SF)

We used the short form of the College Adjustment Scale developed by Lee et al. (2008) to measure the college adjustment level of the participants. The Cronbach’s α of the scale measured in the present study was 0.85.

#### 2.4.9 Korean mental health continuum short form (K-MHC-SF)

We used the Well-being scale of the Korean Mental Health Continuum Short Form to assess participants’ psychological well-being. This scale was developed by Keyes et al. (2008) and translated and validated in Korean by Lim et al. (2012). The Cronbach’s α of the scale measured in the present study was 0.93.

## 3 Results

### 3.1 Composition of preliminary items and preliminary survey

We initially developed 18 preliminary items reflecting promotion or prevention focus. The factor structure of these items was analyzed using Sample 1 (*N* = 215). We applied maximum likelihood with Direct Oblimin rotation due to our expectation of a weak correlation between the promotion-focused and prevention-focused self-consciousness factors (Seo et al., 2018). We identified two factors based on the analysis, eliminating four items (Items 10, 12, 14, 18) with low factor loadings. Consequently, we selected 14 final items, eight related to prevention-focused self-consciousness and six about promotion-focused self-consciousness.

### 3.2 Exploratory factor analysis

Exploratory Factor Analysis was conducted on an independently constructed sample (Sample 2, *N* = 860) to determine the factor structure of the final 14 items of the self-consciousness resulted from the preliminary survey. As in the preliminary survey, we used maximum likelihood extraction and Direct Oblimin rotation method. The KMO suitability index of the sample was 0.896, and Bartlett’s sphericity verification was *X*^2^ = 6772.395 (*p* < 0.001), which was considered suitable for factor analysis.

The result of the analysis confirmed the two-factor structure of the scale, and the two factors explained 61.88% of the total variance. Factor 1 consisted of eight items (2, 3, 6, 7, 9, 11, 15, and 17) reflecting the motivation to minimize experiences that can threaten or feel negatively about oneself. Therefore, factor 1 was named the ‘prevention-focused self-consciousness’ factor. On the other hand, factor 2 consisted of six items (1, 4, 5, 8, 13, and 16) reflecting the motivation to increase the positive experience to move closer to the goal one is pursuing. Therefore, factor 2 was named the ‘promotion-focused self-consciousness’ factor. The prevention focused self-consciousness factor accounted for 35.77% of the total variance, and the promotion-focused self-consciousness factor explained 26.11%. The correlation between the two factors was −0.097, showing a weak but statistically significant negative correlation [*r* (860) = −0.097, *p* < 0.001]. The results of exploratory factor analysis are summarized in Table 2.

**Table 2:** Exploratory factor analysis results of the self-consciousness type scale (Sample 2, *N* = 860).

No.	Question	Factor loading		*M* (SD)
		Factor 1	Factor 2	
*I think about myself a lot. Because…*				
*Factor 1. Prevention-focused self-consciousness (8 questions, α = 0.90)*				
17	I am conscious about what people think of me.	0.89		3.72 (1.33)
6	I am concerned about how others perceive me.	0.86		3.63 (1.36)
2	I care too much what people think.	0.82		3.22 (1.43)
11	It is important to me what others think of me.	0.81		3.84 (1.28)
15	I worry about making mistakes in front of others.	0.71		3.81 (1.36)
9	I do not want to make a bad impression on others.	0.69		4.38 (1.17)
3	I have a lack of confidence.	0.57		3.04 (1.45)
7	I worry that I will do something I regret.	0.54		4.16 (1.31)
*Factor 2. Promotion-focused self-consciousness (6 questions, α = 0.87)*				
16	It helps me understand myself.		0.80	4.84 (1.0)
4	It gives me pleasure to learn about myself.		0.76	4.17 (1.29)
1	I am interested in learning about myself.		0.76	4.53 (1.24)
5	It helps me discover my strength.		0.73	4.40 (1.23)
8	I value self-reflection.		0.67	4.51 (1.21)
13	I need to grow up.		0.67	4.90 (0.98)
*Eigenvalues*		5.007	3.656	
*Percentage of explanation (%)*		35.767	61.880	

*Only questions with a factor loading of 0.4 or higher.

### 3.3 Confirmatory factor analysis

Confirmatory Factor Analysis was conducted on another independent sample (Sample 3, *N* = 1,029) to verify the suitability of the two-factor model derived from the exploratory factor analysis. Because the test result of multivariate normality of the distribution of the data conducted prior to the analysis revealed that it deviated from the assumption of multivariate normality distribution, we conducted a confirmatory factor analysis using the MLM estimation method, one of the robust ML estimators used to analyze non-normal continuous data in Mplus (Muthén and Muthén, 2010).

In order to validate and ensure the reliability of the two sub-factors (promotion- and prevention-focused self-consciousness) identified through confirmatory factor analysis, we calculated the Average Variance Extracted (AVE) and Construct Reliability (CR). The findings revealed that the AVE for the promotion-focused self-consciousness factor was 0.574, with a corresponding CR of 0.890, while for the prevention-focused self-consciousness factor, the AVE was 0.555, with a CR of 0.907. Meeting the established criteria, with values exceeding 0.5 for AVE and 0.7 for CR (Fornell and Larcker, 1981), these results suggest that the items within each sub-factor effectively capture the concepts of promotion- and prevention-focused self-consciousness in a valid and consistent manner. To provide further insight into these findings, we have summarized the AVE and CR values for each factor, alongside standardized factor loadings and standard errors per item, in Table 3.

**Table 3:** Confirmatory factor analysis results, AVE and CR for two subscales of SCTS (Sample 3, *N* = 1,029).

Construct	Items	Standardized factor loadings	S.E.	AVE	CR
Promotion-focused self-consciousness	1	0.796	0.016	0.574	0.890
	4	0.806	0.015		
	5	0.750	0.017		
	8	0.681	0.020		
	13	0.689	0.021		
	16	0.813	0.019		
Prevention-focused self-consciousness	2	0.810	0.014	0.555	0.907
	3	0.590	0.022		
	6	0.876	0.010		
	7	0.674	0.024		
	9	0.662	0.023		
	11	0.822	0.013		
	15	0.681	0.020		
	17	0.873	0.011		

To confirm suitability of the two-factor model, the *Χ*^2^ value and the goodness of fit indices of the model specifically, Comparative Fit Index (CFI), Turcker-Lewis Index (TLI), Root Mean Square Error of Approximation (RMSEA), and Standardized Root Mean Square Residual (SRMR), were comprehensively reviewed. Conventionally, fitness of the model is considered as acceptable when *Χ*^2^ value is not significant, and the goodness of fit indices meet the following criteria: CFI and TLI >0.90, RMSEA<0.08 and SRMR<0.08 (Hu & Hu and Bentler, 1999). In this study, the *Χ*^2^ value of the two-factor model of the self-consciousness was significant at 611.820 (76) but in that *Χ*^2^ value is easily affected by the sample size, we mainly considered CFI, TLI, RMSEA and SRMR to evaluate the model. The RMSEA value showed acceptable suitability with 0.083 (90% confidence interval 0.077, 0.089), CFI and TLI were 0.920 and 0.904, respectively: and SRMR was 0.067, respectively, meeting the criteria for suitability. These goodness-of-fit statistics showed validity of the two-factor model of the newly developed self-consciousness scale in the present study. The results of confirmatory factor analysis are summarized in Table 4.

**Table 4:** Model’s *X*^2^ statistic and goodness-of-fit index (Sample 3, *N* = 1,029).

Model/Fitness Index	$X^{2}$	*df*	RMSEA	90% CI RMSEA	CFI	TLI	SRMR
Observed model	611.820	76	0.083	0.077–0.089	0.920	0.904	0.067

### 3.4 Reliability and validity analysis

#### 3.4.1 Reliability analysis

To confirm the reliability of the self-consciousness type scale, we investigated the internal consistency of the entire scale and the two subscales obtained, as well as examined the correlation between each item and the total score of the subscales. All samples (*N* = 2,104) were used for reliability analysis. Cronbach’s alphas were calculated and found to be 0.79 for the entire scale, 0.88 for the promotion-focused self-consciousness subscale, and 0.91 for the prevention-focused self-consciousness subscale. Considering that the number of items on the scale is not large, it can be regarded as a high level of internal consistency (Nunnally, 1978). The Pearson correlation coefficients between each item and the subscale’s total score were in the range of 0.75 to 0.83 for the promotion-focused self-consciousness subscale and 0.66 to 0.87 for the prevention-focused self-consciousness subscale. The reliability analysis results are summarized in Table 5.

**Table 5:** Reliability analysis of the self-consciousness type scale (*N* = 2,104).

Factor	Question	Mean (Standard deviation)	Correlation with the total
Promotion-focused self-consciousness (6 questions, Cronbach’s *α* = 0.88)	1	4.49 (1.24)	0.83***
	4	4.13 (1.31)	0.83***
	5	4.36 (1.24)	0.79***
	8	4.49 (1.23)	0.75***
	13	4.86 (1.02)	0.75***
	16	4.74 (1.06)	0.83***
Prevention-focused self-consciousness (8 questions, Cronbach’s *α* = 0.91)	2	3.30 (1.45)	0.83***
	3	3.11 (1.46)	0.67***
	6	3.65 (1.37)	0.87***
	7	4.19 (1.33)	0.66***
	9	4.38 (1.20)	0.73***
	11	3.85 (1.28)	0.82***
	15	3.87 (1.34)	0.77***
	17	3.79 (1.32)	0.87***

***p < 0.001, **p < 0.01, *p < 0.05.

#### 3.4.2 Validity analysis

Validity analysis was performed using all samples (*N* = 2,104). First, the correlations between the self-consciousness scale developed in this study and the sub-factors of the SCS (Fenigstein et al., 1975), private self-consciousness, public self-consciousness and social anxiety, were obtained to check its convergent validity as summarized in Table 6. Both the promotion-focused self-consciousness factor [*r*(2,104) = 0.54, *p* < 0.001] and the prevention-focused self-consciousness factor [*r*(2,104) = 0.21, *p* < 0.001] showed a significant correlation with the private self-consciousness factor, which suggests that both factors reflect the tendency of private self-consciousness to pay attention to internal self such as one’s thoughts and emotions. Also, both the promotion-focused self-consciousness factor [*r*(2,104) = 0.13, *p* < 0.001] and the prevention-focused self-consciousness factor [*r*(2,104) = 0.67, *p* < 0.001] were significantly correlated with the public self-consciousness scale. Therefore, both sub-scales reflect the tendency of public self-consciousness to pay attention to oneself socially shown. As for the social anxiety scale, there was a negative correlation with the promotion-focused self-consciousness factor [*r*(2,104) = −0.21, *p* < 0.001], and a positive correlation with the prevention-focused self-consciousness factor [*r*(2,104) = 0.44, *p* < 0.001].

**Table 6:** Correlation between sub-factors of the Self-Consciousness Type Scale and sub-factors of the Self-Consciousness Scale (SCS) (*N* = 2,104).

Scale	1	2	3	4	5	6	7
Prevention focused self-consciousness	1						
Promotion focused self-consciousness	−0.15***	1					
Private self-consciousness (PrSCS)	0.21***	0.54***	1				
Public self-consciousness (PuSCS)	0.67***	0.13***	0.43***	1			
Social anxiety (SCS-SA)	0.44***	–0.21***	0.09***	0.25***	1		
Internal state awareness (PrSCS-ISA)	0.33***	0.31***	0.80***	0.47***	0.17***	1	
Self-reflection (PrSCS-SR)	0.13***	0.57***	0.90***	0.36***	0.03	0.50***	1
*M*	30.14	27.07	32.69	25.88	17.78	10.45	16.24
SD	(8.35)	(5.67)	(5.01)	(4.68)	(5.20)	(2.40)	(3.24)

***p < 0.001, **p < 0.01.

In addition, we investigated the correlations between the newly developed self-consciousness type scale and the sub-factors of the private self-consciousness scale of the SCS, Internal State Awareness (ISA) and Self-Reflectiveness (SR) (Burnkrant and Page, 1984; Watson et al., 1996). The internal state awareness factor was positively correlated with both the prevention-focused factor [*r*(2,104) = 0.33, *p* < 0.001] and the promotion-focused factor [*r*(2,104) = 0.31, *p* < 0.001] of self-consciousness. This finding suggests that both prevention-focused self-consciousness and promotion-focused self-consciousness reflect the process of examining internal conditions such as one’s emotions and body senses at a similar level. On the other hand, even though the self-reflectiveness (SR) factor was positively correlated with both the prevention-focused factor [*r*(2,104) = 0.13, *p* < 0.001] and the promotion-focused factor [*r*(2,104) = 0.57, *p* < 0.001], a significance test using Fisher’s *Z* transformation revealed a significant difference between the correlation coefficients of SR with prevention-focused and promotion-focused self-consciousness (*Z* = −16.75, *p* < 0.001). This suggests that both prevention-focused and promotion-focused self-consciousness tend to evaluate and reflect on oneself overall, and this tendency is higher in promotion-focused self-consciousness than in prevention-focused self-consciousness.

Next, to verify criterion-related validity, we examined the correlation between promotion-focused and prevention-focused self-consciousness factors and depression (CES-D-R), anxiety (GAD-7), self-esteem (RSES), Experience of Shame Scale (ESS), Beck Scale for Suicide Ideation (BSS), perceived stress (PSS), psychological well-being (MHC-SF), and college adjustment (CAI-SF). As a result, prevention-focused self-consciousness showed a significant positive correlation with negative emotions such as depression [*r*(2,104) = 0.34, *p* < 0.001], anxiety [*r*(2,104) = 0.36, *p* < 0.001], and trait shame [*r*(2,104) = 0.58, *p* < 0.001], suicidal ideation [*r*(2,104) = 0.22, *p* < 0.001], and perceived stress [*r*(2,104) = 0.33, *p* < 0.001]. Also, there was a negative correlation with adaptive factors such as self-esteem [*r*(2,104) = −0.41, *p* < 0.001], psychological well-being [*r*(2,104) = −0.26, *p* < 0.001], and college adjustment [*r*(2,104) = −0.27, *p* < 0.001]. On the other hand, promotion-focused self-consciousness showed a significant negative correlation with depression [*r*(2,104) = −0.26, *p* < 0.001], anxiety [*r*(2,104) = −0.17, *p* < 0.001], trait shame [*r*(2,104) = −0.17, *p* < 0.001], suicidal ideation [*r*(2,104) = −0.25, *p* < 0.001], and perceived stress [*r*(2,104) = −0.29, *p* < 0.001], and a positive correlation with self-esteem [*r*(2,104) = 0.42, *p* < 0.001], psychological well-being [*r*(2,104) = 0.39, *p* < 0.001], and college adjustment [*r*(2,104) = 0.28, *p* < 0.001]. These results, summarized in Table 7, reveal consistent associations between prevention- and promotion-focused self-consciousness and adaptive and maladaptive mental-health variables.

**Table 7:** Overall correlations among the self-consciousness type scale, SCS scale, and mental health questionnaire (*N* = 2,104).

Scale	1	2	3	4	5	6	7	8	9	10	11	12	13
Prevention-focused Self-consciousness	1												
Promotion-focused self-consciousness	−0.15***	1											
Private Self-consciousness	0.21***	0.54***	1										
Public Self-consciousness	0.67***	0.13***	0.43***	1									
Social Anxiety	0.44***	−0.21***	0.09***	0.25***	1								
Depression (CES-D)	0.34***	−0.26***	0.16***	0.17***	0.35***	1							
Anxiety (GAD-7)	0.36***	−0.17***	0.22***	0.21***	0.32***	0.80***	1						
Self-esteem (RSES)	−0.41***	0.42***	−0.01	−0.13***	−0.45***	−0.71***	−0.55***	1					
Trait Shame (ESS)	0.58***	−0.17***	0.24***	0.39***	0.44***	0.56***	0.53***	−0.60***	1				
Suicidal ideation (BSS)	0.22***	−0.25***	0.10***	0.07**	0.25***	0.62***	0.51***	−0.62***	0.44***	1			
Perceived Stress (PSS)	0.33***	−0.29***	0.09***	0.15***	0.35***	0.78***	0.67***	−0.68***	0.51***	0.43***	1		
Psychological Well-being (MHC-SF)	−0.26***	0.39***	0.06**	−0.04*	−0.40***	−0.64***	−0.47***	0.72***	−0.41***	−0.50***	−0.66***	1	
College Adjustment	−0.27***	0.28***	−0.04	−0.08**	−0.40***	−0.55***	−0.43***	0.60***	−0.44***	−0.43***	−0.55***	0.58***	1
*M*	30.14	27.07	32.69	25.88	17.78	14.59	4.10	29.53	16.62	7.06	17.43	33.48	53.59
SD	(8.35)	(5.67)	(5.01)	(4.68)	(5.20)	(11.70)	(4.37)	(6.35)	(5.43)	(5.65)	(6.34)	(13.31)	(10.47)

***p < 0.001, **p < 0.01, *p < 0.05.

On the other hand, private and public self-consciousness factors measured using the self-consciousness scale (SCS) showed inconsistent correlations with maladaptive and adaptive mental health indicators. Specifically, private self-consciousness factors showed a positive correlation with maladaptive indicators such as depression [*r*(2,104) = 0.16, *p* < 0.001], anxiety [*r*(2,104) = 0.22, *p* < 0.001], suicidal ideation [*r*(2,104) = 0.10, *p* < 0.001], and perceived stress [*r*(2,104) = 0.09, *p* < 0.001] while also showing a weak positive correlation with psychological well-being [*r*(2,104) = 0.06, *p* < 0.01]. Public self-consciousness factors showed a positive correlation with depression [*r*(2,104) = 0.17, *p* < 0.001], anxiety [*r*(2,104) = 0.21, *p* < 0.001], suicidal ideation [*r*(2,104) = 0.07, *p* < 0.001], and perceived stress [*r*(2,104) = 0.15, *p* < 0.001] and a weak negative correlation with psychological well-being [*r*(2,104) = −0.04, *p* < 0.05] and college adjustment [*r*(2,104) = −0.08, *p* < 0.01].

### 3.5 Cluster analysis and group difference analysis between types of self-consciousness

The two continua model of mental illness and health holds that both are related but distinct dimensions: one continuum indicates the presence or absence of mental health, the other the presence or absence of mental illness. If self-consciousness operates in two independent dimensions, promotion-focused self-consciousness and prevention-focused self-consciousness, the participants may reasonably be classified into four types of self-consciousness on a 2 × 2 model: growth-oriented (high promotion and low prevention), ambivalent (high promotion and high prevention), defensive (low promotion and high prevention), and low-focus (low promotion and low prevention).

**Figure 1:**
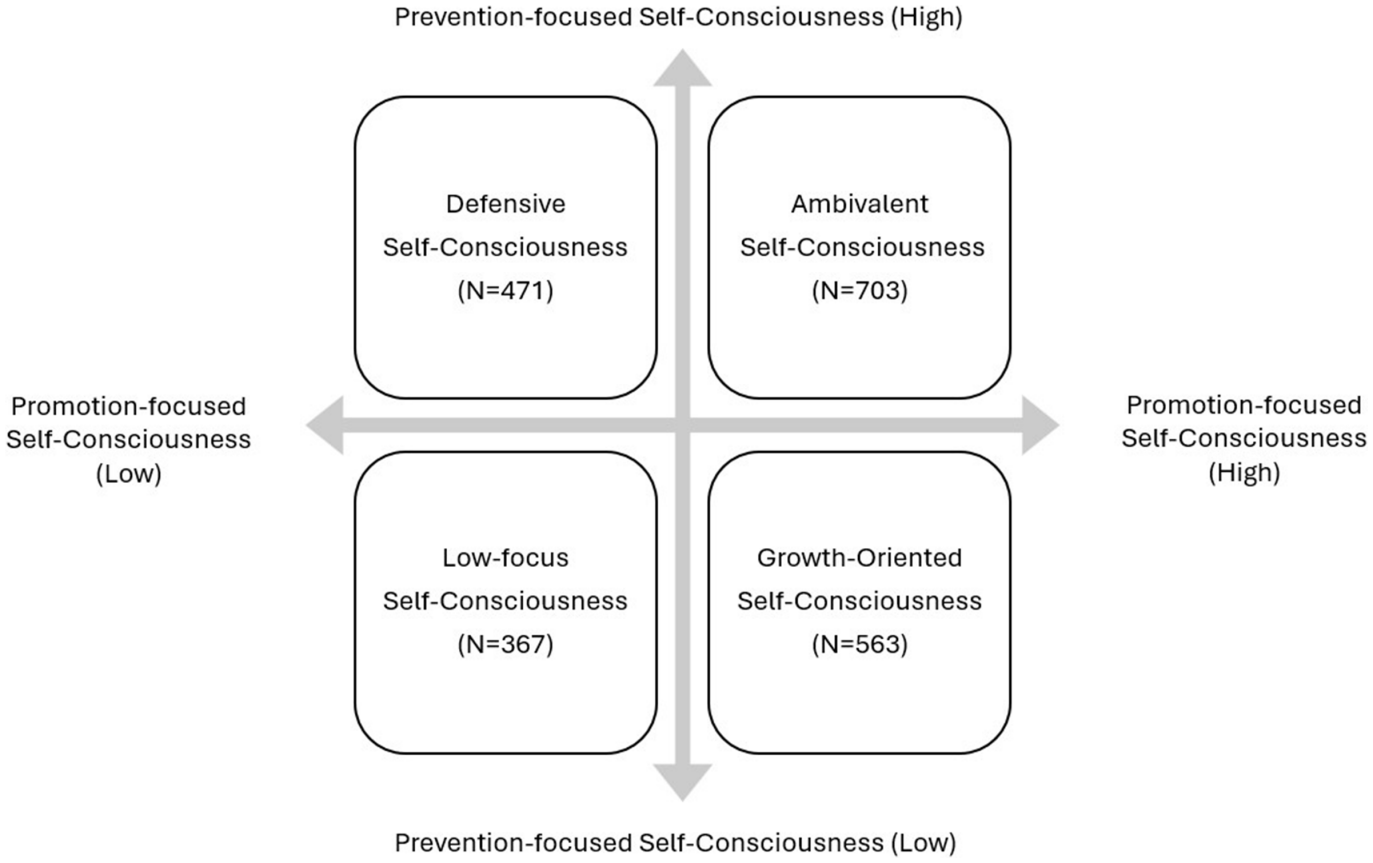
Classification of self-consciousness types.

**Figure 2:**
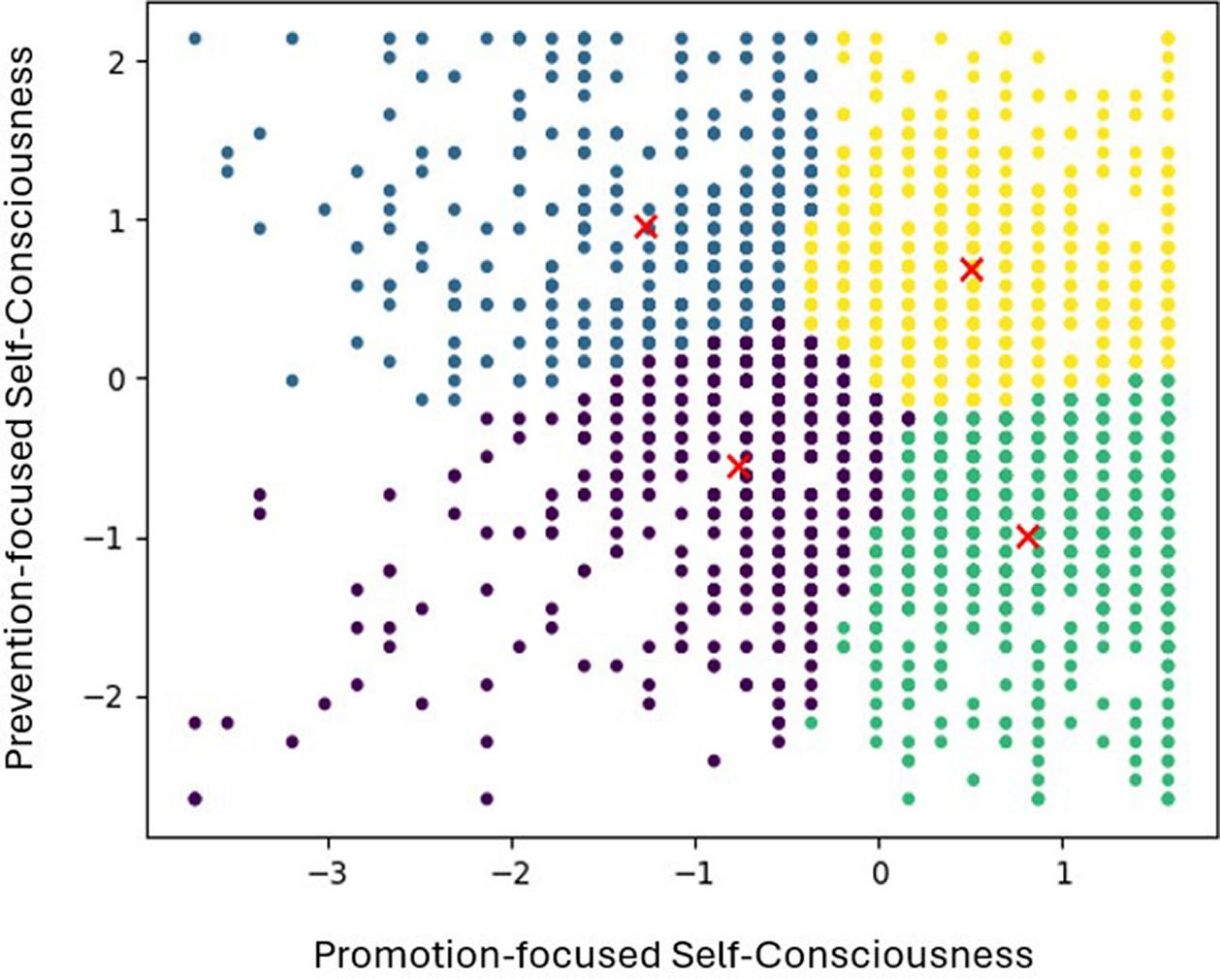
Scatter plot of promotion-focused vs. prevention-focused self-consciousness.

We conducted a k-means cluster analysis in order to categorize the sample (*N* = 2,104) using standardized z-scores of promotion-focused and prevention-focused self-consciousness factors as clustering variables. As a result, we were able to identify four different clusters that differed significantly in both promotion-focused and prevention-focused self-consciousness factors. Specifically, Cluster 1 (*N* = 563, 26.8%) showed highest level of promotion-focused self-consciousness and lowest level of prevention-focused self-consciousness. On the other hand, Cluster 2 (*N* = 471, 22.4%) exhibited the lowest level of promotion-focused self-consciousness and the highest level of prevention-focused self-consciousness. Cluster 3 (*N* = 367, 17.4%) was characterized by showing lower levels in both promotion- and prevention-focused self-consciousness, while Cluster 4 (*N* = 703, 33.4%), which included highest number of participants, exhibited high levels both in promotion- and prevention-focused self-consciousness. Consequently, we named Cluster 1 as the Growth-oriented self-consciousness type, Cluster 2 as the Defensive self-consciousness type, Cluster 3 as the Low-focus self-consciousness type and Cluster 4 as the Ambivalent self-consciousness type. The results are summarized and presented in Table 8 and Figure 1. Additionally, Figure 2 displays a scatter plot of promotion-focused versus prevention-focused self-consciousness scores, with data points colored according to the four identified clusters. Cluster centroids, marked with red Xs, indicate the central tendencies of each cluster. Specifically, Cluster 1 (Growth-Oriented) is represented by green, Cluster 2 (Defensive) by blue, Cluster 3 (Low-Focus) by purple, and Cluster 4 (Ambivalent) by yellow. The plot illustrates distribution of the two SCTS subscales and the distinct patterns of self-consciousness types within the sample.

**Table 8:** Self-consciousness types identified through cluster analysis (*N* = 2,104).

Variables	Cluster 1 (*n* = 563,26.8%)	Cluster 2 (*n* = 471,22.4%)	Cluster 3 (*n* = 367,17.4%)	Cluster 4 (*n* = 703,33.4%)	*F*-value
	***M* (SD)**				
Promotion-focused self-consciousness	31.52(2.71)	20.97(3.96)	22.25(4.14)	30.12(2.93)	1286.097***
Prevention-focused self-consciousness	21.84(5.00)	36.80(5.00)	23.72(5.15)	35.67(4.60)	1338.654***

***p < 0.001.

After classifying the participants into four groups based on the 2 × 2 model of self-consciousness, a one-way ANOVA and Scheffe’s post-hoc comparison analysis were conducted to determine whether there were significant differences among the groups regarding mental health and adaptation-related variables (i.e., depression, anxiety, suicidal ideation, trait shame, self-esteem, perceived stress, college adjustment, and psychological well-being). Significant differences (*p* < 0.001) were found among the four groups in all measurements with each variable showing a slightly different pattern.

The results of ANOVA and post-hoc comparison analysis are summarized and presented in Table 9. First, all four groups had a significant difference in anxiety and depression. Specifically, the highest anxiety scores were found in the defensive self-consciousness group, followed by the ambivalent self-consciousness group, the low-focus self-consciousness group, and the growth-oriented self-consciousness group. Similarly, significant differences were observed in depression scores among the four groups. The defensive self-consciousness group had the highest scores, followed by the ambivalent self-consciousness group, the low-focus self-consciousness group, and the growth-oriented self-consciousness group. The measurements of suicidal ideation and perceived stress revealed a common tendency: the defensive self-consciousness group scored the highest, and the growth-oriented self-consciousness group scored the lowest; there was no significant difference between the ambivalent and low-focus self-consciousness groups. The shame measurement was also the highest in the defensive self-consciousness group, followed by the ambivalent self-consciousness group. Growth-oriented and low focus self-consciousness groups showed significantly lower shame scores than ambivalent self-consciousness group. Lastly, in the case of adaptation-related variables represented by self-esteem, college adjustment, and psychological well-being, the growth-oriented self-consciousness group scored the highest, the defensive self-consciousness group scored the lowest, and the ambivalent self-consciousness and low-focus self-consciousness groups showed no significant difference. The effect size (η^2^) of the group differences was also examined, with all variables showing values of 0.06 or higher. According to Cohen’s (1988) criteria, this indicates a medium or larger effect size. This suggests that self-consciousness types account for a significant portion of the variation in mental health-related variables. Notably, the effect sizes for shame and self-esteem were 0.14 or higher, indicating large effect sizes. Since shame is a representative self-conscious emotion and self-esteem directly reflects attitudes toward the self, it can be interpreted that self-consciousness types explain a larger portion of these variables. Considering the above findings, we named this newly developed self-consciousness scale the “Self-consciousness Type Scale.”

**Table 9:** Group differences in Mental Health Questionnaire scores among different Self-consciousness types (*N* = 2,104).

Scale	Self-consciousness types				*F*-value	Effect size ( $\eta^{2}$ )	*Post hoc* test (Scheffe)
	Growth-Oriented Self-Consciousness (High promotion – Low prevention) (*n* = 563)	Defensive Self-Consciousness (Low promotion – High prevention) (*n* = 471)	Low-Focus Self-Consciousness (Low promotion – Low prevention) (n = 367)	Ambivalent Self-Consciousness (High promotion – High prevention) (*n* = 703)			
	*M* (SD)						
Anxiety	2.39 (3.20)	5.72 (4.74)	3.40 (4.22)	4.76 (4.47)	63.979***	0.08	2 > 4 > 3 > 1
Depression	9.50 (8.89)	20.33 (12.55)	13.37 (11.66)	15.46 (11.23)	85.088***	0.11	2 > 4 > 3 > 1
Suicidal ideation	5.51 (4.52)	9.46 (6.87)	6.74 (5.45)	6.85 (5.13)	45.875***	0.06	2 > 3, 4 > 1
Perceived stress	14.38 (5.94)	20.73 (5.85)	17.16 (6.29)	17.80 (5.81)	99.104***	0.12	2 > 3, 4 > 1
Trait shame	13.53 (3.63)	19.83 (5.72)	14.29 (4.27)	18.16 (5.16)	201.924***	0.22	2 > 4 > 1, 3
Self-esteem	33.35 (4.77)	25.07 (6.23)	29.68 (5.71)	29.38 (5.89)	183.160***	0.21	1 > 3, 4 > 2
College adjustment	57.86 (9.65)	48.68 (10.31)	53.87 (9.91)	53.33 (10.01)	72.690***	0.09	1 > 3, 4 > 2
Psychological well-being	39.90 (12.79)	25.92 (12.23)	32.63 (12.81)	33.84 (11.91)	109.848***	0.14	1 > 3, 4 > 2

***p < 0.001.

## 4 Discussion

Self-consciousness plays a pivotal role in the self-regulation process and constitutes a significant variable influencing mental health and psychological adaptation. Concurrently, self-consciousness manifests a duality, demonstrating pathological aspects by inducing negative emotions or acting as a common factor in various psychopathologies. Grounded in the regulatory focus theory, the present study aimed to measure self-consciousness operating under distinct regulatory foci, namely promotion focus and prevention focus. The objective was to develop a self-report scale that validly measures two different dimensions of self-consciousness, promotion-focused and prevention-focused self-consciousness, and to utilize each dimension to categorize individuals into four self-consciousness types. Additionally, the study sought to examine whether the four identified subtypes exhibit significant differences in mental health-related variables.

The summary of the research findings is as follows. Firstly, exploratory factor analysis was conducted on the selected 14 items of the final Self-Consciousness Type Scale (SCTS), resulting in the identification of two factors: promotion-focused self-consciousness and prevention-focused self-consciousness. Subsequently, confirmatory factor analysis was performed to assess the structural validity of the scale, demonstrating a satisfactory level of goodness-of-fit indices. Although the correlation between the two sub-factors was somewhat low, it was statistically significant. This finding contrasts with the results of Higgins et al.’s (2001) study, where the correlation between promotion and prevention focus factors was not statistically significant. However, in contrast to the exclusive focus on motivation in Higgins et al.’s (2001) study, the SCTS includes certain common elements of self-consciousness in both promotion-focused and prevention-focused self-consciousness. Therefore, the observed low correlation between promotion-focused and prevention-focused self-consciousness seems reasonable considering these differences.

To assess the convergent validity of the self-consciousness type scale, correlations were computed with the most widely used consciousness scale, the Self-Consciousness Scale (SCS: Fenigstein et al., 1975). The results indicated that both promotion-focused and prevention-focused self-consciousness exhibited significant positive correlations with both private and public self-consciousness factors. This suggests that both promotion and prevention-focused self-consciousness comprehensively reflect a tendency to direct attention inwardly toward the internal self and external self. Furthermore, to validate criterion-related validity, the study examined the correlations between the promotion-focused and prevention-focused self-consciousness factors and variables related to mental health and psychological adaptation. The findings revealed that promotion-focused self-consciousness exhibited positive correlations with self-esteem, psychological well-being, and adaptation to university life, and negative correlations with depression, anxiety, suicidal ideation, shame, and perceived stress. In contrast, prevention-focused self-consciousness showed negative correlations with self-esteem, psychological well-being, and adaptation to university life, and positive correlations with depression, anxiety, suicidal ideation, shame, and perceived stress.

These results suggest that promotion-focused self-consciousness tends to be more closely associated with the adaptive variables, while prevention-focused self-consciousness appears to be more closely related to the maladaptive variables. This is consistent with findings from Ouyang et al.’s (2015) study, where individuals with promotion-focused tendencies reported higher subjective well-being compared to those with prevention-focused tendencies. It also aligns with McGregor et al.’s (2007) study, which demonstrated a positive correlation between self-esteem and dispositional promotion focus, and a negative correlation with dispositional prevention focus. Moreover, considering that promotion-focus showed no significant correlation with rumination, while prevention-focus exhibited a significant correlation in the same study, it can be inferred that the prevention-focused self-consciousness factor in the present study might reflect the characteristic of rumination, considered a dysfunctional form of self-consciousness (Trapnell and Campbell, 1999).

Finally, we classified the participants into four self-consciousness types through cluster analysis, using promotion and prevention-focused self-consciousness as two clustering variables. Subsequently, a group difference analysis was conducted to ascertain if there were significant differences in mental health-related metrics among these types. The group characterized by high promotion-focused self-consciousness and low prevention-focused self-consciousness was named the Growth-oriented Self-consciousness Type, while the group with low promotion-focused self-consciousness and high prevention-focused self-consciousness was termed the Defensive Self-consciousness Type. When both promotion- and prevention-focused self-consciousness were high, the group was designated as the Ambivalent Self-consciousness Type, and the group with low levels of both was labeled the Low-focus Self-consciousness Type. Significant differences among the groups were observed in all measured variables, with anxiety and depression showing particularly significant differences across the four types. Particularly noteworthy were the significant differences in anxiety among the four types, with the Defensive Self-consciousness Type displaying the highest anxiety scores, followed by the Ambivalent Self-consciousness Type, the Low- focus Self-consciousness Type, and the Growth-oriented Self-consciousness Type in descending order. This aligns with the proposition by Higgins et al. (1997) that the frustration of prevention focused goals triggers anxiety.

Meanwhile, in the case of shame, it was most pronounced in the Defensive Self-consciousness Type, followed by relatively high scores in the Ambivalent Self-consciousness Type. No significant differences were observed between the Growth-oriented Self-consciousness and Low-focus Self-consciousness Types. This aligns with the argument proposed by Pounders et al. (2018) that individuals experiencing shame tend to focus on their deficient aspects, are sensitive to negative outcomes, and exhibit avoidance coping tendencies, thereby being closely associated with prevention focus. In terms of suicidal thoughts, and perceived stress, the Defensive Self-consciousness Type exhibited the highest scores, while the Growth-oriented Self-consciousness Type showed the lowest scores. No significant difference was found between the Low- focus Self-consciousness Type and the Ambivalent Self-consciousness Type. In the case of self-esteem, university life adaptation, and psychological well-being, the Growth-oriented Self-consciousness Type significantly demonstrated the highest values, whereas the Defensive Self-consciousness Type exhibited the lowest values. Although no significant statistical differences were found between Low-focus Self-consciousness and Ambivalent Self-consciousness Types, the overall well-being scores were higher in the Ambivalent Self-consciousness Type than in the Low-focus Self-consciousness Type. This is consistent with the findings of Lee and Kwon’s (2005) study, where individuals with higher general self-focused attention but lower dysfunctional self-consciousness, such as self-immersion, named ‘non-defensive self-focused attention’ group, exhibited higher self-esteem and lower anxiety compared to the group with low self-focused attention.

Taken together, the results of this study suggest the existence of multiple dimensions of self-consciousness showing qualitative differences, rather than supporting a unidimensional model of self-consciousness that posits a simple excess leading to psychological maladaptation. This supports a multidimensional model wherein each dimension exerts distinct influences on mental health. Additionally, the study revealed that the motivation behind thinking about oneself and directing attention, rather than the extent or specific aspects of such self-focus (e.g., private self and public self), plays a crucial role in psychological adaptation.

The present study has the following limitations. Firstly, as it relies on self-report measures, there is a possibility that participants’ meta-cognitive abilities, response tendencies, or social desirability might have influenced the results. Therefore, future research would benefit from incorporating supplementary methodologies to assess the validity of responses, including clinician-administered interviews, social desirability scales, and meta-cognition assessments. Additionally, it would be necessary to investigate whether participants consistently demonstrate individual differences in self-consciousness by examining correlations with implicit and behavioral measures of self-consciousness, such as word association tasks (Eichstaedt and Silvia, 2003) or sentence completion tasks (Wegner and Giuliano, 1980). Secondly, the scale developed in the study was validated with undergraduates and graduates at a university in Seoul; thus, there are limitations in generalizing the research findings. Subsequent studies should administer the scale to diverse age groups and populations to conduct additional validation work. Thirdly, a limitation arises from relying solely on participants’ self-reported responses as indicators of psychological adaptation and maladaptation. It would help if future studies incorporates more objective and various indicators of mental health. Additionally, studying clinical samples could provide valuable insights into the prevalence of different self-consciousness types among groups with various clinical conditions. This could include not only groups with depression or anxiety disorders, but also conditions that are not addressed in the present study, such as bipolar disorder, narcissistic personality disorder, and impulse control problems. Lastly, the cross-sectional study design and correlational analysis utilized in this study does not allow for the determination of the directionality or causality between self-consciousness styles and psychopathology. Future research should longitudinally examine how individuals with various self-consciousness types respond and adapt to different life stressors as in Suls and Fletcher’s (1985) study.

Despite these limitations, the present study holds significance for the following reasons. Firstly, it provides a tool for distinguishing different types of self-consciousness and demonstrates their relationship with mental health and adaptation related variables. Since prevention-focused self-consciousness and promotion-focused self-consciousness are independent dimensions showing continuity within an individual, the combination of these dimensions yields four types of self-consciousness, and significant differences among the types were identified in psychological adaptation-related and maladjustment-related measures. This approach of categorizing self-consciousness into four types using the two continuous dimensions is expected to enhance the utility of the scale compared to merely utilizing two factor scores of promotion-focused and prevention-focused self-consciousness. This can offer important implications for designing mental health programs tailored to the characteristics of each group.

Secondly, it was an attempt to move beyond the classification of public and private self-consciousness and measure the inclination to think about and pay attention to oneself in an integrated manner. The validity of the conceptual distinction between public and private self-consciousness has been questioned several times (e.g., Wicklund and Gollwitzer, 1987; Schlenker and Weigold, 1990). However, existing scales that aimed to distinguish between the adaptive and maladaptive aspects of self-consciousness (e.g., RRQ, SRIS, SDSAS) accepted only the private self-consciousness factor for differentiation. In contrast, the present scale is significant in its attempt to integrate both factors without distinguishing between private and public self-consciousness. In fact, within the SCTS, both prevention-focused and promotion-focused self-consciousness exhibited significant positive correlations with both public and private self-consciousness simultaneously.

Thirdly, when compared to Fenigstein et al.’s (1975) Self-Consciousness Scale (SCS), commonly used in previous research, the SCTS developed in the present study demonstrated higher explanatory power for mental health-related variables than the SCS. Specifically, the private/public self-consciousness factors measured using the SCS in the study showed correlations with both adaptive and maladaptive indicators, and the magnitudes of these correlations were notably weak. In contrast, the SCTS exhibited generally stronger correlations with mental health-related variables than the SCS. Promotion-focused self-consciousness was consistently correlated with adaptive indicators, while prevention-focused self-consciousness demonstrated consistent correlations with maladaptive indicators. Furthermore, in comparison to the SCS, which primarily reflects maladaptive aspects of self-consciousness, the SCTS holds significance for validly reflecting not only the maladaptive aspects but also the healthy aspects of self-consciousness.

Finally, the attempt to identify different types of self-consciousness in terms of motivational aspects can have implications for psychotherapy. Since chronically self-focused attention has a biological basis (Morin, 2011a,b) and involves unconscious and implicit processes, interventions aimed at reducing or eliminating self-focused attention itself may not be effective, contrary to Ingram’s (1990) suggestion. Therefore, it is essential to consider shifting the focus of intervention towards adaptively altering cognitive factors such as motivation, context, and interpretation, which direct attention to oneself based on the multidimensional nature of self-consciousness, rather than attempting to reduce self-consciousness itself. From this perspective, proposing that there could be a more adaptive dimension to cognitive motivation, involving thinking about and paying attention to oneself, is more valid from the perspective of cognitive therapy and aligns with the principles of positive psychology as well (Kim and Ko, 2009). Instead of viewing self-consciousness as a risk factor or vulnerability that needs to be eliminated, accepting it as a neutral and natural personal trait and learning to use self-consciousness adaptively can lead to more positive outcomes. This finding further suggests that cultivating a healthy self-consciousness contributes not only to the absence of psychological distress but also to the pursuit of well-being in life.

## Data availability statement

The raw data supporting the conclusions of this article will be made available by the authors, without undue reservation.

## Ethics statement

The studies involving humans were approved by Korea University Institutional Review Board (KUIRB-2022-0206-02). The studies were conducted in accordance with the local legislation and institutional requirements. The participants provided their written informed consent to participate in this study.

## Author contributions

JL: Writing – original draft, Writing – review & editing. HB: Writing – review & editing. EO: Investigation, Project administration, Writing – review & editing. J-yK: Conceptualization, Supervision, Writing – review & editing. Y-gK: Conceptualization, Data curation, Formal analysis, Funding acquisition, Investigation, Methodology, Project administration, Resources, Supervision, Validation, Writing – review & editing.
